# Treatment of dementia: recommendations of the Scientific Department of Cognitive Neurology and Aging of the Brazilian Academy of Neurology

**DOI:** 10.1590/1980-5764-DN-2022-S106PT

**Published:** 2022-11-28

**Authors:** Paulo Caramelli, Valeska Marinho, Jerson Laks, Marcus Vinicius Della Coletta, Florindo Stella, Einstein Francisco Camargos, Jerusa Smid, Breno José Alencar Pires Barbosa, Lucas Porcello Schilling, Marcio Luiz Figueredo Balthazar, Norberto Anízio Ferreira Frota, Leonardo Cruz de Souza, Francisco Assis Carvalho Vale, Márcia Lorena Fagundes Chaves, Sonia Maria Dozzi Brucki, Ricardo Nitrini, Helen Bedinoto Durgante, Paulo Henrique Ferreira Bertolucci

**Affiliations:** 1 Universidade Federal de Minas Gerais, Faculdade de Medicina, Departamento de Clínica Médica, Belo Horizonte MG, Brasil.; 2 Universidade Federal do Rio de Janeiro, Instituto de Psiquiatria, Centro para Doença de Alzheimer, Rio de Janeiro RJ, Brasil.; 3 Universidade Federal do Rio de Janeiro, Instituto de Psiquiatria, Programa de Pós-Graduação em Psiquiatria e Saúde Mental, Rio de Janeiro RJ, Brasil.; 4 Universidade do Estado do Rio de Janeiro, Faculdade de Ciências Médicas, Rio de Janeiro RJ, Brasil.; 5 Universidade do Estado do Amazonas, Manaus AM, Brasil.; 6 Universidade Estadual Paulista, Instituto de Biociências, Campus de Rio Claro SP, Brasil.; 7 Universidade de São Paulo, Faculdade de Medicina, Departamento e Instituto de Psiquiatria, Laboratório de Neurociências, São Paulo SP, Brasil.; 8 Hospital Universitário de Brasília, Centro de Medicina do Idoso, Brasília DF, Brasil.; 9 Universidade de Brasília, Faculdade de Medicina, Programa de Pós-Graduação em Ciências Médicas, Brasília DF, Brasil.; 10 Universidade de São Paulo, Faculdade de Medicina, Departamento de Neurologia, Grupo de Neurologia Cognitiva e do Comportamento, São Paulo SP, Brasil.; 11 Universidade Federal de Pernambuco, Centro de Ciências Médicas, Área Acadêmica de Neuropsiquiatria, Recife PE, Brasil.; 12 Instituto de Medicina Integral Prof. Fernando Figueira, Recife PE, Brasil.; 13 Pontifícia Universidade do Rio Grande do Sul, Escola de Medicina, Serviço de Neurologia, Porto Alegre RS, Brasil.; 14 Pontifícia Universidade do Rio Grande do Sul, Instituto do Cérebro do Rio Grande do Sul, Porto Alegre RS, Brasil.; 15 Pontifícia Universidade do Rio Grande do Sul, Programa de Pós-Graduação em Gerontologia Biomédica, Porto Alegre RS, Brasil.; 16 Universidade Estadual de Campinas, Departamento de Neurologia, Faculdade de Ciências Médicas, Campinas SP, Brasil.; 17 Hospital Geral de Fortaleza, Serviço de Neurologia, Fortaleza CE, Brasil.; 18 Universidade de Fortaleza, Fortaleza CE, Brasil.; 19 Universidade Federal de São Carlos, Centro de Ciências Biológicas e da Saúde, Departamento de Medicina, São Carlos SP, Brasil.; 20 Hospital de Clínicas de Porto Alegre, Serviço de Neurologia, Porto Alegre RS, Brasil.; 21 Universidade Federal do Rio Grande do Sul, Faculdade de Medicina, Departamento de Medicina Interna, Porto Alegre RS, Brasil.; 22 Universidade Federal de Pelotas, Pelotas RS, Brasil.; 23 Universidade Federal de São Paulo, Escola Paulista de Medicina, Departamento de Neurologia e Neurocirurgia, São Paulo SP, Brasil

**Keywords:** Dementia, Drug Therapy, Behavior, Cognition, Demência, Tratamento Farmacológico, Comportamento, Cognição

## Abstract

There is currently no cure for neurodegenerative or vascular dementias, but some pharmacological and non-pharmacological interventions may contribute to alleviate symptoms, slow disease progression and improve quality of life. Current treatment approaches are based on etiology, symptom profile and stage of dementia. This manuscript presents recommendations on pharmacological and non-pharmacological treatments of dementia due to Alzheimer’s disease, vascular cognitive impairment, frontotemporal dementia, Parkinson’s disease dementia, and dementia with Lewy bodies.

## RESUMO

Atualmente não há tratamento curativo para as demências neurodegenerativas ou para a demência vascular, mas algumas intervenções farmacológicas e não farmacológicas podem contribuir para aliviar os sintomas, retardar a progressão da doença e melhorar a qualidade de vida. As abordagens terapêuticas atuais são baseadas na etiologia, no perfil dos sintomas e no estágio da demência. Neste artigo apresentamos recomendações sobre os tratamentos farmacológicos e não farmacológicos da demência devida à doença de Alzheimer, comprometimento cognitivo vascular, demência frontotemporal, demência da doença de Parkinson e demência com corpos de Lewy.

## INTRODUCTION

Research estimates that by 2050 about 152 million people will have dementia, or one new case every three seconds, mostly from low- and middle-income countries (LMIC). Currently, about two thirds of people diagnosed with dementia (PwD) live in LMIC^1^, added to a high estimate of underdiagnosed cases-approximately 800.000 people in Brazil-according to recent data^2^.

Dementia is one of the leading causes of disability in older adults, with significant impacts on the autonomy and quality of life (QoL) of PwD and their families. Neurodegenerative diseases and cerebrovascular disorders are the main causes of dementia, with Alzheimer’s disease (AD) accounting for more than half of the cases^3^. In Brazil, AD ranked among the top ten causes of deaths in 2019^4^. Other degenerative causes include frontotemporal dementia (FTD), Parkinson’s disease (PD) dementia (PDD) and dementia with Lewy bodies (DLB).

Although we currently lack a cure for neurodegenerative or vascular dementias, some pharmacological and non-pharmacological interventions can contribute to alleviate symptoms, slow disease progression and improve QoL^5^. Therapeutic approaches are based on etiology, symptom profile and stage of dementia. Thus, this manuscript presents updated information and recommendations on current available treatments in Brazil for dementia due to AD, vascular cognitive impairment (VCI), FTD, PDD and DLB.

## TREATMENT OF DEMENTIA DUE TO AD

### Pharmacological treatment

Degeneration of basal forebrain cholinergic neurons precedes clinical manifestations in AD, thus implying an early deficit in cholinergic activity in mild to moderate stages of dementia due to AD^6^. Cholinesterase inhibitors (ChEI) donepezil, galantamine, and rivastigmine inhibit the ChE enzyme and increase acetylcholine availability in the brain. These drugs are approved for treating mild and moderate AD dementia, but have also shown positive effects in severe stages^7^. For moderate-to-severe AD dementia, studies recommend combining ChEI with the glutamatergic NMDA receptor antagonist memantine.

Despite modest effects, use of ChEI and memantine have shown improvement in disease symptoms or slower progression when compared with placebo. Clinical benefits can be seen in cognitive, behavioral, and functional domains^5^ ^),(^ ^7^. Despite conflicting evidence, research shows that all ChEI may reduce AD mortality and galantamine may reduce the risk of conversion to severe dementia^8^.

**Figure 1:**
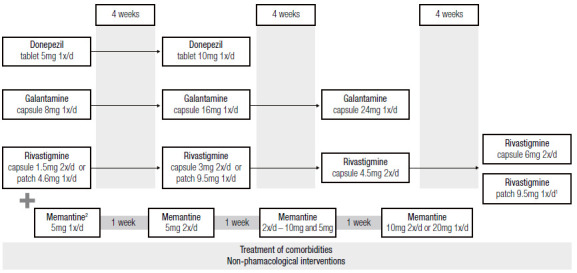
Overview of pharmacological treatment for AD dementia. ^1^In case of improvement or stabilization, the highest dose of rivastigmine patch can be postponed; ^2^memantine is indicated for treating moderate or severe AD dementia.

When prescribing these drugs, physicians must clarify to PwD and caregivers that the expected clinical effects are usually modest, sometimes characterized only by stabilization or slower worsening. Therapy should begin with the lowest dosage to avoid adverse events (AEs) and be increased at least every four weeks for ChEI and one week for memantine. Regarding ChEI, usage should reach the therapeutic doses, i.e., 5-10mg for donepezil, 16-24 mg for galantamine, 6-12mg for oral rivastigmine and 9.5mg for rivastigmine patch (the 13.3mg patch is also available and can be prescribed for selected cases). Ideally, the highest dose within the therapeutic range of each drug should be targeted, given the greater potential for clinical benefits. Figure 1 illustrates the dose regimen for drug therapy of AD dementia.

Common AEs consist of nausea, vomiting, diarrhea, headache and insomnia, and are less likely to occur if medication is taken after a meal. Donepezil should be administered at bedtime; in case of insomnia or nightmares, it can be taken in the morning. Less common, but more serious AEs are bradyarrhythmia and syncope. Treatment effects and duration vary from person to person, and any positive effect may be lost over time. No research has confirmed the superiority of one ChEI over the others, despite some specificities on the mechanisms of action and frequency/types of AEs. Switching to another ChEI is encouraged, particularly when no clinical benefit is observed after a few months of follow-up or in case of AEs that do not resolve. With progression to moderate dementia, the association with memantine is indicated^5^ ^),(^ ^9^ ^),(^ ^10^. Memantine is usually well tolerated; the most common AEs are drowsiness, dizziness and headache^5^ ^),(^ ^7^.

### Practical issues for prescribing and discontinuing ChEI and memantine

- ChEI increase vagal tone and are contraindicated in patients with baseline bradycardia or known cardiac conduction system disorder (i.e., atrioventricular block > 1^st^ degree). Caution is required when any of the three ChEI are used in combination with drugs that induce bradycardia or alter atrioventricular conduction (e.g., beta blockers or calcium channel blockers);
- Caution is required when prescribing ChEI to individuals with chronic obstructive pulmonary disease or asthma;
- Discontinuation or switching (in the case of ChEI) shall be considered when there is no clinical benefit or in case of relevant AEs;
- Avoid abrupt discontinuation of ChEI or memantine;
- In the case of AEs with higher doses, dose reduction may be considered, noting whether there is a decrease in AEs as well as changes in cognitive, functional, or neuropsychiatric symptoms;
- Discontinue medication in the absence of clinical benefits or if AEs persist.

### Pharmacological treatment of neuropsychiatric symptoms

Neuropsychiatric symptoms may precede the cognitive symptoms in AD, becoming more common at the second half of the mild dementia stage, increasing along the moderate stage and usually decreasing at severe stage.

Clinical trials specifically addressing neuropsychiatric symptoms in AD have been disappointing. The benefits of ChEI tend to favor specific neuropsychiatric symptoms, such as depression, anxiety, dysphoria, and apathy^11^. For depression, which may be present in more than half of AD patients, there is some evidence for the indication of sertraline or mirtazapine as the best antidepressant options^12^. A recent clinical trial, however, observed a trend to increased mortality with mirtazapine used to treat agitation in AD dementia^13^.

Aggressiveness is a common neuropsychiatric symptom which may contribute to agitation and increased caregiver burden. Treatment encompasses both pharmacological and non-pharmacological interventions (see next session). Specific AD drugs (i.e., ChEI and memantine) might have modest but significant effects on these symptoms and even if there is a good initial effect, it may be lost after some time. Given the absence of approved pharmacological treatment for neuropsychiatric symptoms in AD dementia, a sequence of drugs may be considered for treatment of agitation and aggressiveness^14^. According to this algorithm^14^, first-line treatment would be atypical antipsychotics, the first choice being risperidone, followed by aripiprazole or quetiapine. If these drugs fail or are offset by AEs, carbamazepine may be an option. In case of a new failure the next step would be citalopram, followed by gabapentin and prazosin. Citalopram is a good therapeutic option for agitation. Benzodiazepines must be avoided in dementia due to AD.

Another common neuropsychiatric symptom is sleep disorder. The first step to a successful treatment of sleep disorder in AD is to ask why and how it occurs. A sleep disorder may be the consequence of a sleep wake cycle inversion, and in this case sleep hygiene and non-pharmacological interventions may be helpful. For pain or another discomfort, the cause should be addressed; issues related to sundowning, fragmented sleep or difficulty in starting sleep should be addressed. The sleep may be interrupted by delusions and hallucinations, when an antipsychotic, particularly with sedative effects (e.g., quetiapine) may be useful. There is no evidence that hypnotics or mirtazapine have positive effects. Trazodone might improve sleep, although the effects are usually modest^15^. A recent Brazilian study has shown that short-term use (i.e., 14 days) of zopiclone or zolpidem may be clinically helpful in treating insomnia in AD dementia^16^.

Delusions and hallucinations may be present in up to one third of AD patients and indicate poorer prognosis, with increased functional impairment and higher mortality. Treatment of these neuropsychiatric symptoms includes avoiding typical antipsychotics, due to AEs and cognitive worsening. There is evidence of a modest effect of ChEI, but often there is a need of using antipsychotics, and in this case the evidence is stronger for risperidone and olanzapine, while for quetiapine the evidence is scanter. Studies with brexpiprazole are ongoing, but evidence of efficacy and safety has not been definitively established to date^17^. Although some studies suggest that cannabinoids may have potential positive effects in neurodegenerative diseases, there is no current evidence to support their clinical use in dementia^18^.

### Pharmacological treatment for AD-related conditions

AD may have associated complications. Parkinsonism may be present in 9% to 70%, depending on the disease stage. There are differences with typical PD, with tremor being mild or absent, and usually presenting symmetrical bradykinesia and rigidity from the start. Levodopa may be tried, but response is less likely than in PD.

Between 10% and 20% of AD patients will have at least one seizure. Newer anticonvulsants (e.g., lamotrigine or levetiracetam) are first-line treatments for epilepsy in PwD^19^.

### Non-pharmacological treatments for AD: cognitive symptoms

#### Cognitive stimulation therapy (CST)

Recommended treatment according to the National Institute for Health and Clinical Excellence of the United Kingdom^20^ to stimulate language, memory, executive functions; approved by the Alzheimer’s Disease International and used in many countries^21^. CST was shown to improve cognition, QoL, neuropsychiatric symptoms in PwD from LMIC^22^ ^),(^ ^23^. CST and its implementation manual have been adapted for use in Brazil^24^ and the intervention was found to be feasible and beneficial to improve mood in PwD in a recent clinical trial^25^.

#### Multicomponent/multidisciplinary cognitive rehabilitation program (MCRP)

MCRP includes cognitive rehabilitation, stimulation and computer-assisted therapy, physical training, physiotherapy, reading and games. This program may decrease depressive symptoms and improve QoL indicators of PwD/caregivers. However, MCRP effects on cognition are questionable^23^ ^),(^ ^26^.

#### Reality orientation (RO)

Neurosensory stimulation to reorient daily activities may improve cognition and adherence to pharmacological treatment in mild-moderate AD dementia^27^ ^),(^ ^28^. Results for neuropsychiatric symptoms are inconclusive^29^.

### Non-pharmacological treatments for AD: neuropsychiatric symptoms

To increase the chance of success with a non-pharmacological treatment it is essential to adopt an individualized approach that considers:

- When and how the neuropsychiatric symptom started? (Was there a trigger?)
- How did it evolve? (Are there associated symptoms?)
- How did it end?
- What are the associated worsening and improving factors?

#### Psychosocial/psychoeducational interventions

These are group or person-centered approaches to inform the management and care of dementia, including emotional and social stimulation of PwD/caregivers. Face-to-face or technology-based multicomponent psychoeducational and Cognitive-Behavioral Therapy approaches (cognitive reframing, self-monitoring, mood management techniques, mindfulness-based training) may improve neuropsychiatric symptoms (agitation, restlessness, anxiety, sleep disturbances), delay the progression of cognitive impairment, and benefit caregiver confidence, self-efficacy, burden, depression, stress levels, and QoL of the dyad^30^ ^)- (^ ^33^. Positive Psychology practices are recommended for mental health promotion of PwD/caregivers according to the Alzheimer’s Association Psychosocial Measurement Workgroup^33^ and the European INTERDEM Social Health Taskforce^34^ ^),(^ ^35^.

#### Reminiscence therapy (RT)

RT is a group-based intervention to recall autobiographical memories. It is used for mild-moderate dementia with modest evidence to improve depressive symptoms, mood, cognition^22^, and well-being^36^. Lacking theoretical basis and methodological systematization make RT effects questionable^37^.

#### Music therapy (MT)/dancing

Significant positive effects were found of MT on memory^38^, emotional/mood^22^ and neuropsychiatric symptoms^27^. Dancing may also lead to beneficial effects on cognition and psychical health^20^.

#### Physical-related/lifestyle-related interventions

Repetitive transcranial magnetic stimulation (rTMS) and acupuncture seem to improve cognitive functions, whereas acupuncture, exercise and light therapy presented potential to enhance functional performance^22^ and cognition^39^ ^),(^ ^40^. Long-term exercise improves blood flow, hippocampal volume, neurogenesis, also neuropsychiatric symptoms^41^. Occupational therapy (error-reduction techniques) ^(^ ^42^ and physiotherapy^43^ ^),(^ ^44^ tend to enhance functioning and delay physical decline; results are inconclusive for improvement in cognition. Early interventions targeting lifestyle modifiable risk factors (arterial hypertension, diabetes, obesity, physical inactivity, high alcohol consumption, smoking, social isolation), clinical conditions (hearing loss and depression), and poor nutrient intake, may work as protective measures for reducing AD risk^1^ ^),(^ ^10^ ^),(^ ^45^ ^),(^ ^46^.

## TREATMENT OF VASCULAR COGNITIVE IMPAIRMENT

The general principles of approach and treatment for VCI are the same as for dementia due to AD. They encompass the treatment of comorbidities, including neuropsychiatric symptoms, providing information and support to PwD and caregivers, and maintaining independence.

### Primary and secondary prevention

The multidisciplinary team should focus primarily on controlling the main risk factors for new cerebrovascular events and cognitive impairment in VCI. Although we recognize risk factors for post-stroke dementia, such as low education and diabetes mellitus^47^, factors for stroke itself have long been known and controllable, including smoking, arterial hypertension, diabetes mellitus and dyslipidemia (notably high LDL-cholesterol levels) ^(^ ^48^.

However, in recent decades several studies have proposed measuring the weight of these risk factors on the appearance of new strokes, on post-stroke cognition and functionality, often with conflicting results. Based on these observations we recommend:

- Blood pressure control: There is strong evidence that increased blood pressure levels are associated with stroke^48^. There are still no studies that have confirmed the impact of blood pressure control on worsening cognitive impairment. Intensive blood pressure control (systolic pressure of 120 *versus* 140 mmHg) does not show a reduction in the incidence of dementia (all etiologies). In frail older adults, with symptoms of orthostatic hypotension and increased risk of falls, individualize blood pressure targets to avoid symptoms of hypotension;
- Glycemic control: There is strong evidence that increased blood glucose levels are associated with stroke^48^. However, there is no evidence that strict glycemic control in older adults prevents cardiovascular events. Studies have shown that tight blood glucose control in older adults was associated with greater frailty and mortality, with no benefit in the evolution of dementia^49^ ^),(^ ^50^. Acceptable fasting glycemic values in individuals with VCI should be around up to 150 mg/dL and postprandial < 180 mg/dL, as well as glycated hemoglobin targets should be less stringent (e.g., < 8%)^51^;
- Antiplatelet agents: There is strong evidence to support using these agents for secondary prevention of non-embolic stroke, either acetylsalicylic acid (81 to 100mg/day) or clopidogrel (75mg/day) ^(^ ^52^. In cases of VCI without evidence of previous stroke, therapy should be individualized^53^, especially in individuals with significant risk of falls. In recent years, no studies have been published providing support for the benefits of antiaggregants in the evolution of VCI^54^;
- Statins: Statins are of interest in the secondary prevention of cerebrovascular disease due to both their lipid-lowering and pleiotropic effects on vascular function, combining to inhibit atherosclerosis. There is good evidence that statins given late in life to people at risk for vascular disease do not prevent cognitive decline or dementia^55^. For individuals with VCI and a history of ischemic (non-embolic) stroke, statin use should follow secondary prevention recommendations with individual risk analysis. The use of statins in older adults and subjects with vascular risk factors is not recommended for the sole purpose of primary prevention or treatment of dementia;
- Multidisciplinary interventions: In a systematic review of 15 prospective studies with over 30,000 individuals without dementia, involvement in at least one physical activity of minimal to moderate intensity was associated with a 35% reduction in the relative risk of cognitive decline in one to 12 years of follow-up^56^. In another study with 639 older adults without disability and with cerebral white matter changes, physical activity reduced the risk of cognitive and functional deficits, regardless of the severity of white matter changes or other risk factors^57^. Hence, practice of physical activity at mild to moderate intensity should be strongly recommended to all individuals at the primary care level as a means of primary and secondary prevention of VCI. On the other hand, evidence that physical rehabilitation reduces post-stroke cognitive loss is still insufficient^58^;
- Diet and supplements: A balanced diet, rich in vegetables and fruits, impacts many organ systems. One recent systematic review with metanalysis has shown that high adherence to the Mediterranean diet reduced the risk of global cognitive decline in non-demented older adults^59^. The use of vitamin and mineral supplements should only be indicated in cases with insufficiency. It is not recommended to use vitamin supplements for the prevention or treatment of VCI.

### Pharmacological treatment

#### ChEI and memantine

The pharmacological treatment commonly prescribed in VCI, particularly vascular dementia, is mainly based on ChEI and memantine. These medications are routinely used in clinical practice to treat cognitive decline, functionality changes and neuropsychiatric symptoms. However, randomized controlled clinical trials have shown limited efficacy of these drugs.

Randomized, placebo-controlled studies demonstrate little efficacy of ChEI in pure vascular dementia^60^ ^),(^ ^61^. There is a statistically significant but clinically slight cognitive benefit for cognitive functions, mainly processing speed and executive functions^62^. In general, there is a slight attenuation of cognitive decline compared to placebo^61^. Some studies report favorable results regarding functional performance, but the gain is not consistent. Improvement of neuropsychiatric symptoms, when present, is also limited. On the other hand, agitation has been observed as an AE of ChEI and memantine^63^ ^),(^ ^64^.

Favorable results of ChEI seem more evident in mixed dementia (vascular + AD) compared to ‘pure’ vascular dementia^65^. Galantamine is approved in Brazil for treatment of mild to moderate AD dementia with concomitant significant cerebrovascular disease and has shown positive effects on cognitive speed and QoL of individuals with mixed dementia in a controlled clinical trial in the country^66^. Possible benefits of ChEI in mixed dementia, although limited, are attributed to the reduction of cholinergic depletion caused by concomitant AD^60^ ^),(^ ^65^ ^),(^ ^66^. There is insufficient clarity regarding the differences in efficacy of ChEI, as well as memantine, between multiple infarcts and subcortical vascular dementia^64^ ^),(^ ^67^ ^)-(^ ^69^. Initiation of ChEI treatment in patients with severe dementia is not recommended.

Among the AEs of ChEI already described above, bradycardia and arterial hypotension are the main conditions associated that require special attention in patients with vascular dementia^62^ ^),(^ ^65^ ^),(^ ^67^ ^)-(^ ^71^.

In current clinical practice, memantine is commonly added to ChEI in subjects with moderate or severe vascular dementia. However, the benefits of memantine are very limited or controversial for controlling cognitive and neuropsychiatric symptoms, as well as for slowing cognitive or functional decline^61^. For this reason, the drug has not been approved for treatment of vascular dementia. The few randomized, placebo-controlled studies report that memantine is well-tolerated and that drowsiness has been the AE of major concern^72^.

### Treatment of neuropsychiatric symptoms in VCI

Overall, the neuropsychiatric symptoms in VCI are like those observed in AD, and the treatment is similar^14^ ^),(^ ^73^ ^)-(^ ^75^. The non-pharmacological interventions include occupational therapy, cognitive stimulation, art therapy, involvement in social interaction activities, physical activity, and psychological support for PwD and caregivers.

Regarding pharmacological interventions, general recommendations follow the commentaries made above (AD section), namely, to avoid benzodiazepines, to prescribe antipsychotics in low doses and for strictly necessary periods, antidepressants for depression, anxiety, panic and mild agitation, and anticonvulsants when indicated.

The pharmacological treatment of patients with vascular dementia should be regularly monitored for cardiovascular signs and symptoms.

## TREATMENT OF FRONTOTEMPORAL DEMENTIA (BEHAVIORAL AND LANGUAGE VARIANTS) 

Frontotemporal dementia (FTD) encompasses different clinical syndromes associated with progressive behavioral/personality deterioration or language disorders, and is considered the second most frequent cause of young-onset dementia^76^.

### Treatment of behavioral variant frontotemporal dementia

The clinical syndrome associated with behavioral and personality changes called behavioral variant (bvFTD) is the most common clinical presentation and specific diagnostic criteria are available for clinical use^77^.

There are no approved treatments for bvFTD, either disease-modifying drugs or symptomatic treatments to ameliorate disturbing symptoms. Only a few small studies have shown benefits of symptomatic pharmacological approaches^78^.

Considering that there are no specific treatments, care should focus on managing behavioral symptoms and disability, and reducing caregiver burden that is high in bvFTD. In a recent Brazilian study including 20 bvFTD and 30 AD individuals, Lima-Silva et al. found out that bvFTD subjects presented higher levels of neuropsychiatric symptoms and that their caregivers experienced higher levels of distress than AD caregivers. Moreover, bvFTD caregiver’s distress and burden were related to cognitive and functional impairment^79^.

Multidisciplinary management may be needed throughout the disease process, but this has not been specifically established for bvFTD treatment. A multidisciplinary team may offer a more comprehensive care management and seems to be an essential tool for dealing with other degenerative diseases^80^. However, the needs in bvFTD may be more specific and studies regarding their management should be conducted. Social, financial, psychological counseling may be required to address specific issues and more knowledge on those topics should be gathered to help services’ organization. For instance, the emergence of swallowing problems during dementia progression may require specialized evaluation. Problems with inappropriate eating speed, passivity, coughing, and choking beginning in the mild stages and following the worsening cognitive and behavioral declines were recently reported and add information about specific treatment needs^81^.

Regarding pharmacological approaches, a recent systematic review gathered information aiming to identify pharmacological interventions that could be used to treat the more troublesome behaviors in bvFTD. Trieu et al. ^(^ ^78^ found 23 studies (11 randomized controlled trials, eight open-label studies, one proof-of-concept study, and three case series) involving a sample of 573 individuals. Sixteen out of the 23 studies used pharmacological interventions, resulting in benefits, as measured by the Neuropsychiatric Inventory, for trazodone, citalopram, rivastigmine, paroxetine, ameliorating symptoms such as disinhibition, hyperorality, and depression.

Two reviews^82^ ^),(^ ^83^ published by Brazilian authors report that most studies have small sample sizes, short duration of treatment, and non-uniform measures while evaluating efficacy and tolerability.

Portugal et al. ^(^ ^82^ conducted a systematic review and reported better results for drugs with serotonergic action, such as selective serotonin reuptake inhibitors or SSRIs (paroxetine, citalopram, fluvoxamine) and trazodone to treat behavioral symptoms, but not cognition. Authors suggest that using SSRIs as the first-line treatment seems to be the best practice until better data on evidence-based strategies to rely upon are available.

In a narrative review aiming to guide clinical practice, Gambogi et al. ^(^ ^83^ conducted a literature search on bvFTD treatment. According to drug class, ChEI and memantine do not have significant therapeutic actions. Antidepressants (SSRIs and trazodone) improved behavioral symptoms with better tolerability; antipsychotics reduced agitation (quetiapine, risperidone, aripiprazole), improper behaviors (risperidone and aripiprazole), and scores on the Neuropsychiatric Inventory (olanzapine) in uncontrolled cases series and clinical reports. However, mortality issues related to antipsychotic use in dementia, cardiovascular risks, and motor AEs limit their use in bvFTD. Psychostimulants for apathy and anticonvulsants for abnormal behaviors were investigated, but must be used with caution due to AEs. In a symptom-focused approach, authors suggest SSRIs or trazodone as the first option for disinhibition, compulsive/perseverative behaviors, stereotyped behaviors, and hyperorality. Psychostimulants may be an option for apathy and antipsychotics should be used with care and reserved to manage challenging and hazardous behaviors.

In summary, several low-quality reports are focusing on symptomatic treatment for bvFTD and no specific treatment proved to be effective to date. ChEI and memantine should not be used as they have not shown any significant therapeutic action. SSRIs or trazodone seem the best practice to manage behavioral problems as a first step, and the use of second-generation antipsychotics should be reserved for the management of psychotic manifestations, troublesome or hazardous behaviors.

### Treatment of primary progressive aphasias

Primary progressive aphasia (PPA) refers to clinical syndromes characterized by an insidious and progressive loss of language abilities associated with neurodegenerative diseases. The three variants differ in anatomic, phenotypic presentation, and underlying pathology^84^.

The non-fluent/agrammatic variant PPA (nfvPPA) is associated with atrophy of left frontal lobe, insula, and supplementary motor areas, and progressive, effortful, non-fluent speech, with syntactic deficits, grammatical errors, and omissions. The semantic variant PPA (svPPA) is associated with left anterior temporal lobe atrophy and fluent speech with deficits in lexical retrieval, naming, and word comprehension. The logopenic variant PPA (lvPPA) presents AD as the pathological substrate in most cases and is associated with left posterior temporal/parietal atrophy, and slow speech with long-word finding pauses, anomia, and repetition deficits^84^.

Up to this moment, there are no disease-modifying agents approved for these language syndromes associated with neurodegenerative diseases. Symptomatic treatments aiming to improve language disorders have been shown to enhance oral and written naming abilities^85^. Language training therapies improve naming accuracy for trained items at short- and long-term follow-up and are considered the standard treatment for PPA^85^ ^),(^ ^86^.

There are very few studies on PPA treatment in Brazil and Latin-America, with small sample sizes, demonstrating the lack of local information about language therapies in PPA^87^. Notwithstanding, an important piece of information was gathered by a Brazilian systematic review organized by Carthery-Goulart et al. ^(^ ^88^. The literature on evidence-based strategies of language rehabilitation in PPA was summarized and the language behavioral treatments classified into impairment-directed and functional interventions. The results suggest, as a practical option, the recommendations for the use of impairment-directed therapies aimed at naming and lexical retrieval in svPPA. In relation to nfvPPA and lvPPA, the small number of studies limits conclusions about the best therapeutic option for such variants^88^.

While impairment-directed intervention targets the remediation or slowing the progression of specific language and speech impairments, the functional interventions focus on communication, including environmental modifications. The functional interventions key components rely on building communication strategies and practicing the strategies with a communication partner. However, the frequency and dosage of such interventions using rigorous outcomes is an unmet need up to this moment^89^.

The maintenance of therapy gains, in the absence of continuous training, is more evident in the short-term than long-term in all PPA variants and is influenced by treatment length and session frequency. Generalization to untreated items varies following each PPA subtype and occurs more often in nfvPPA and lvPPA than in svPPA^90^.

Language therapies usually focus on enhancing abilities in trained activities or tasks and such training may limit benefits to the practiced domains^86^ ^),(^ ^88^. Recent reviews have shown a potential benefit for non-invasive brain stimulation combined with language therapies in improving oral or written naming accuracy for trained and untrained items^85^ ^),(^ ^86^.

As a practical orientation cognitive-linguistic interventions targeting impairment and aiming at remediation and symptoms’ improvement seem the best practice. In this sense, a recent Brazilian study investigated the short- and mid-term effects of four training programs directed to language and speech deficits in 18 PPA individuals (different subtypes) carried out during four months (two weekly sessions). All cases improved performance on trained items during the active phase of treatment. Statistically significant clinical benefits were observed in 13 individuals, while for five, the results were maintained. However, generalization to untrained items or to other tasks were observed only in two individuals^91^.

## TREATMENT OF PARKINSON’S DISEASE DEMENTIA AND DEMENTIA WITH LEWY BODIES

PD and DLB are neurodegenerative disorders that share some common features such as dopaminergic depletion and movement disorders^92^. Lewy body inclusions throughout the cortex ensue the typical triad of the dementia syndrome in DLB: dementia, complex visual hallucinations and parkinsonism. PDD, in turn, starts with a dysexecutive syndrome^93^ ^),(^ ^94^.

DLB may show parkinsonism later in the course of the disease, whereas complex visual hallucinations appear early, together with *delirium* without other clinical causes. PD first symptoms, on the other hand, encompass asymmetric bradykinesia, tremor and muscle rigidity^95^. Memory complaints are not the first symptoms in DLB or in PDD, as usually observed in dementia due to AD and VCI. PDD typically presents with a frontal dysexecutive syndrome^95^ ^),(^ ^96^.

The identification of the disease which is causing dementia is important to identify drugs that should and should not be prescribed when neuropsychiatric symptoms occur. Antipsychotics should be avoided in DLB, as there is a hypersensitivity to neuroleptic malignant syndrome and because they may aggravate parkinsonism, regardless of the dose prescribed. When psychosis exists in PDD, levodopa and other antiparkinsonian drugs should be re-evaluated, before prescribing any antipsychotics to deal with the behavioral and mood symptoms^97^ ^),(^ ^98^.

Cognitive and neuropsychiatric symptoms in PDD and DLB are treated with rivastigmine, the only approved ChEI for these indications^98^ ^),(^ ^99^. Memantine appears to be associated with some improvement in behavioral aspects, but studies have not reported benefits for cognition in these individuals^98^ ^),(^ ^99^. Memantine has not been approved for treatment of DLB or PDD.

As for treatment of neuropsychiatric symptoms and syndromes, stimuli regulation should be tried before any medication is applied. Excessive stimulation, or the lack of proper stimulation, can worsen orientation and behavior. Room light, physical exercise, cognitive stimulation, calm environment and maintenance of clinical status can help control behavioral problems before medication comes into place^100^. Even if medication is needed, all these measures should be enforced.

The clinical diagnosis, differentiating PDD from DLB, can be a challenge, even for the specialist, since the two conditions seem to be different spectra of the same pathological family, being mainly differentiated by the clinical course.

The cortical and subcortical distribution of Lewy bodies will lead to different clinical presentations. Although it seems somewhat theoretical, this distinction is essential in some details of the medication management inherent to each situation. Diagnostic criteria for PDD and DLB have been published before^96^ ^),(^ ^97^.

### Lewy body diseases spectrum

**Figure 2:**
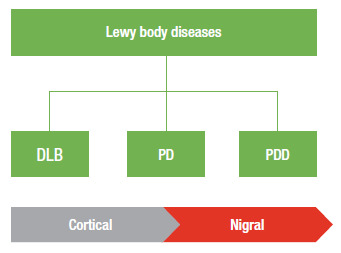
Topographic distribution pattern of Lewy bodies in dementia with Lewy bodies (DLB), Parkinson’s disease (PD) and dementia associated with Parkinson’s disease (PDD).

Due to the inherent characteristics of PDD and DLB, several clinical-pathological correlation studies include the two conditions as different spectra of the same pathological process. In the present consensus, we consider the two diseases from this viewpoint, i.e., different involvements of a common pathological substrate. In PDD, the accumulation of Lewy bodies is mainly concentrated in subcortical areas, while in DLB the pathological process affects cortical areas (Figure 2) ^(^ ^101^ ^)-(^ ^103^.

### Management of DLB

There is no specific disease-modifying treatment for DLB. Non-pharmacological and pharmacological interventions can be used in symptomatic management. There are no specific non-pharmacological interventions in DLB^100^ ^),(^ ^104^.

#### Motor symptoms

Levodopa is the drug of choice to treat motor symptoms. Slow titration is important to achieve some motor response with minimal psychiatric AEs. Response to levodopa in DLB can be limited, especially in the later stages, due to the predominance of levodopa non-responsive symptoms. Dopamine agonists are not recommended due to the risk of worsening hallucinations. Anticholinergics are contraindicated^97^ ^)-(^ ^99^ ^),(^ ^105^ ^),(^ ^106^.

#### Neuropsychiatric symptoms

The use of antipsychotics must be done very judiciously, due to the high sensitivity of these patients, with important paradoxical reactions^107^. Typical antipsychotics are contraindicated. Atypical antipsychotics, specifically quetiapine and clozapine, can be used, always under close supervision, to detect AEs^97^ ^),(^ ^99^ ^),(^ ^108^. Of special note, clozapine may cause neutropenia in about 3% of cases, which demands weekly monitoring of blood cell count within the first 18 weeks of treatment.

As for ChEI (mainly rivastigmine), besides the cognitive benefits several studies have shown positive effects on neuropsychiatric symptoms, particularly visual hallucinations, delusions, and apathy. It is essential to be aware that the withdrawal of ChEI, for any reason, may be associated with cognitive deterioration^99^ ^),(^ ^109^ ^)-(^ ^111^.

## CONCLUSIONS

Although current treatments for neurodegenerative and vascular dementias are only symptomatic, several evidenced-based therapies are available and may provide benefits to Pwd and to their caregivers. The clinician must combine the best pharmacological options with the best available non-pharmacological interventions, taking into consideration the underlying etiology, symptoms profile and severity of the dementia syndrome, as well as the clinical and personal history of each individual.

It is important to inform the PwD and their families about the potential expected benefits of the overall treatment, as well as the possible AEs of the prescribed drugs. Regular follow-up is critical (ideally every three to four months in the beginning of treatment and around every six months later on) to evaluate the clinical effects and whether adjustments (e.g., drug dosing, additional pharmacological and non-pharmacological interventions) are needed.

Given that the modest effects of the available treatments and the lack of approved disease-specific drug therapies, prevention of dementia is a key principle. Several modifiable risk factors have been identified and lifestyle and clinical interventions (e.g., adequate control of hypertension and diabetes, regular physical activity, diet programs, cognitive stimulation activities) shall be recommended for middle-age and older adults to reduce the risk of cognitive impairment and dementia, thus contributing to mitigate the personal, socioeconomic and public health impacts of these devastating disorders. ^(^ ^45^
